# Reconsidering Granulocyte Infusions in Prolonged Neutropenia: A Case Report

**DOI:** 10.7759/cureus.113173

**Published:** 2026-07-22

**Authors:** Ananya Murali, Glenn Seela, Markie Zimmer, Vijayalakshmi Donthireddy

**Affiliations:** 1 Hematology and Medical Oncology, Henry Ford Health System, Detroit, USA; 2 Internal Medicine, Henry Ford Health System, Detroit, USA

**Keywords:** acute myeloid leukemia (aml), granulocyte colony-stimulating factor (g-csf), granulocyte-macrophage colony-stimulating factor, granulocyte transfusion, severe neutropenia

## Abstract

A 39-year-old man with therapy-related acute myeloid leukemia (t-AML) had refractory disease after induction with 7+3 (cytarabine for seven days and daunorubicin for three days). Reinduction with FLAG-Ida (fludarabine, cytarabine, G-CSF, and idarubicin) resulted in a hypocellular bone marrow (<5%) and prolonged pancytopenia complicated by mucormycosis. He received granulocyte colony-stimulating factor (G-CSF) and granulocyte-macrophage colony-stimulating factor (GM-CSF) without improvement, leading to the decision to implement granulocyte infusions. There was temporary and mild improvement in the neutrophil count with resolution of mucormycosis. Ultimately, the patient succumbed to refractory AML. This case highlights the potential use of granulocyte infusions for prolonged neutropenia with infectious complications, while addressing the logistical difficulties and transient benefit of the intervention.

## Introduction

Neutropenia, defined by an absolute neutrophil count (ANC) of less than 1.5 × 10^9^/L (or 1,500 cells/µL), is often caused by hematologic malignancies and related chemotherapy [1]. The degree and duration of neutropenia determine the clinical impact. Often, the only presenting sign of neutropenia is fever, which warrants a detailed evaluation and prompt management [2].

Granulocyte colony-stimulating factor (G-CSF) is commonly used as primary or secondary prophylaxis to temporarily increase the absolute neutrophil count (ANC) until myelosuppression resolves. Prophylactic antibacterial and antifungal regimens must be considered [3]. Granulocyte-macrophage colony-stimulating factor (GM-CSF) use can also be employed. While both G-CSF and GM-CSF stimulate the production of granulocytes, their use varies based on the severity of neutropenia. In the realm of AML, GM-CSF is typically used after trying G-CSF, as it targets neutrophils, monocytes, and eosinophils, while the latter only targets the neutrophil lineage. If neutropenia is recalcitrant to G-CSF and GM-CSF, a hematopoietic stem cell transplant may be considered [4].

Literature surrounding granulocyte infusions is limited. The majority of current evidence suggests that they can act as a temporary rescue against life-threatening infectious complications. One study showed that there can be an approximate ANC increase of 1 × 10^9^/L maintained for only 1-1.5 days, emphasizing their transient effect [5]. In this regard, an infusion may simply be described as a bridge that allows the patient time to mount their own immune response against the infection. A case study done by the NIH discussed the effect of granulocyte infusions on 11 patients affected by invasive fusariosis, a fungal infection resistant to many antifungal therapies. While the study showed that 10 of the 11 patients survived for at least 30 days after the infusion, only 5 of the 11 patients were discharged from the hospital alive. Thus, granulocyte infusions are a temporizing measure, and addressing the underlying etiology of neutropenia is paramount [6,7].

Although granulocyte infusions open an exciting avenue for research and patient care, it would be remiss to not mention the associated risks. The most common adverse effects are fever and chills, allo-immunization, and pulmonary complications, ranging from hypoxemia and pulmonary infiltrates to a life-threatening transfusion-related acute lung injury (TRALI). Literature reports a 25%-50% incidence of mild to moderate adverse effects and a 1% incidence of severe adverse effects and suggests that slower infusion rates may ameliorate the risk of some of these adverse effects [6].

## Case presentation

A 39-year-old man with a past medical history of metastatic seminoma in remission after a left-sided orchiectomy, chemotherapy with etoposide and cisplatin, and four cycles of paclitaxel, ifosfamide, and cisplatin developed high-risk myelodysplastic syndrome with a 5q deletion and 13q gain. He was ineligible for allogeneic stem cell transplantation due to heart disease and end-stage renal disease. He was treated with azacitidine and venetoclax but had disease progression to AML by cycle 3 of azacitidine and venetoclax. He was started on a dose-modified 7+3 regimen, consisting of daunorubicin for three days and cytarabine for seven days, to account for the low glomerular filtration rate. The day 14 bone marrow biopsy demonstrated persistent blasts, prompting initiation of reinduction chemotherapy with the FLAG-Ida regimen (fludarabine, cytarabine, G-CSF, and idarubicin). Repeat bone marrow biopsy revealed hypocellular marrow (<5%) with no evidence of leukemia.

**Figure 1:**
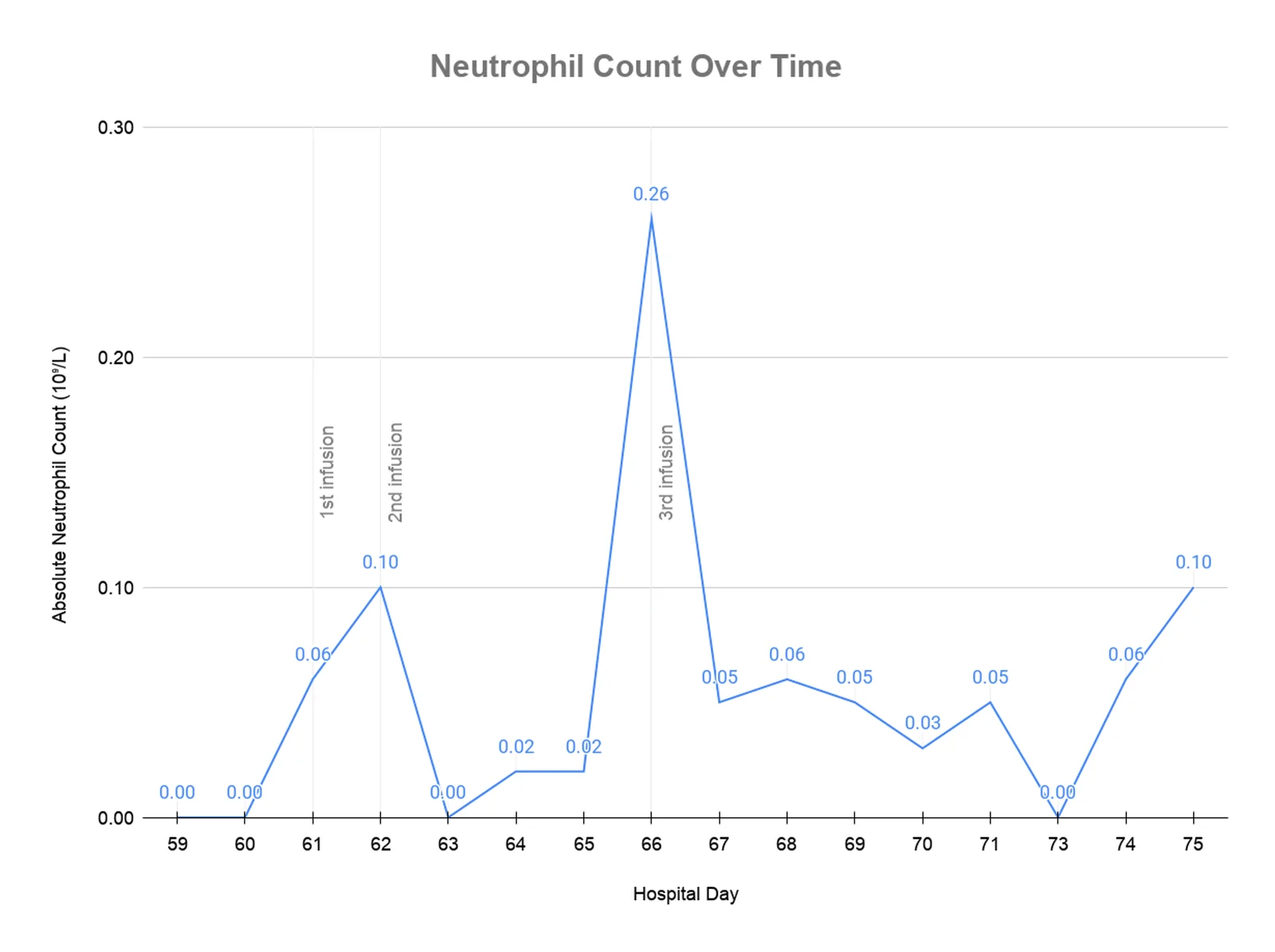
Neutrophil Count Over Time

He had prolonged severe neutropenia, and on day 53 following initiation of FLAG-Ida, he developed nasal congestion and sinus pain with a two-day fever. CT of the head and sinuses demonstrated paranasal sinus disease with air-fluid levels in the right maxillary and frontal sinuses. Nasal biopsy confirmed angioinvasive and necrotizing fungal elements, with pathology confirming mucormycosis. This prompted the initiation of 1,000 mg amphotericin B every 24 hours and surgical debridement. He was initiated on tbo-filgrastim (a recombinant form of G-CSF) with an undetectable neutrophil count after two weeks of therapy. Therapy was changed to sargramostim (a recombinant form of GM-CSF), which also failed to produce any significant improvement, with ANC fluctuating between 0 and 0.05 × 10^9^/L for 17 days before discontinuation due to futility, as shown in Table 1 and Figure 1. In light of the decreased white blood cell (WBC) and ongoing fungal sinusitis requiring debridement, a decision was made to treat with three total granulocyte infusions based on the current literature. The duration and number of sessions rely on the clearance of infection, response to treatment, and logistical aspects of the transfusion process. While the number of sessions can range anywhere between three days and months, one major randomized controlled trial showed patients receiving a median of five transfusions before cessation [8,9]. For our patient, each granulocyte infusion was accompanied by Tylenol 975 mg, Benadryl 50 mg, hydrocortisone 100 mg, and Demerol 12.5 mg prior. ABO-compatible, irradiated granulocyte products were administered starting at a slow infusion rate of 2 mL/min and increased to infuse over a three-hour duration if the infusion is tolerated.

**Table 1:** Hospital Course With Recorded Absolute Neutrophil Count

Hospital Day (During Most Recent Hospitalization)	Absolute Neutrophil Count (K/uL)	Notes
59	0.00	
60	0.00	
61	0.06	1st Granulocyte Infusion
62	0.10	2nd Granulocyte Infusion
63	0.00	
64	0.02	
65	0.02	
66	0.26	3rd Granulocyte Infusion
67	0.05	
68	0.06	
69	0.05	
70	0.03	
71	0.05	
73	0.00	
74	0.06	
75	0.10	

Our patient tolerated ongoing GM-CSF therapy and all three infusions, with no signs of respiratory distress or fever. After receiving the third granulocyte infusion, his ANC increased temporarily to 0.26 × 10^9^/L, as demonstrated in Table 1 and Figure 1, but ultimately decreased to 0.0-0.10 × 10^9^/L. While the improvement in neutropenia was short-lived, it can be argued that the granulocyte infusions decreased his time to fungal clearance. A repeat endoscopy the day after completing the granulocyte infusions revealed complete resolution of the mucormycosis.

## Discussion

The use of granulocyte infusions remains controversial with limited supporting data. Our case study emphasizes granulocyte infusions as a supportive treatment option for critically ill patients with neutropenia. While there remains some controversy in individual cases, proposed indications for granulocyte infusion in the literature generally include severe neutropenia (ANC < 0.5 × 10^9^/L), confirmed or suspected fungal or bacterial infection unresponsive to antimicrobial therapy, and an anticipated recovery in neutrophil counts following transfusion [6].

Notably, granulocyte infusions are not without limitations. It is imperative to recognize that granulocyte infusions do not address the underlying etiology; they are a temporizing measure, and the underlying etiology needs to be addressed [5]. Additionally, healthcare teams should be aware of the challenges of obtaining granulocytes from a donor using appropriate storage and transporting techniques, as they must be stored at a temperature of 22°C in order to preserve their functionality [8]. Granulocytes tend to drastically lose their effect shortly after collection, and therefore, same-day infusions are critical [10]. Additionally, it is imperative to find suitable donors to provide the granulocytes, an additional safety measure that may take days, preventing the patient from receiving a timely transfusion [11]. The biggest limitation to the use of granulocyte infusions is argued to be the lack of research surrounding the use of granulocytes to address infections in neutropenic patients. Current literature is largely based upon individual case reports and case series [11]. As limited, and sometimes conflicting, research surrounds their use, guidelines are needed to delineate which patients are appropriate candidates for granulocyte infusions and provide information regarding safe handling, transfer, and storage of granulocytes in addition to protocols to manage infusion reactions. Lastly, the question has been posed whether consideration for granulocyte infusions may become obviated with further advances in antimicrobial therapy, including novel antifungal agents, prophylactic strategies in AML, immunomodulators, and donor-derived cellular therapies, to prevent life-threatening infections in the midst of severe neutropenia [6].

## Conclusions

This case highlights the potential role of granulocyte infusions as supportive therapy in severe neutropenia. While our patient received the transfusions without an adverse event, their use is limited by accessibility, risk of transfusion reactions, and non-durable responses without modifying the underlying disease. Further reporting of granulocyte infusion administration will better define the appropriate clinical context for their use.
